# Supplementary material for: A novel motorized office chair causes low-amplitude spinal movements and activates trunk muscles: A cross-over trial
Source: PLoS One. 2023 Dec 22;18(12):e0294778. doi: 10.1371/journal.pone.0294778 (PMC10745177; doi:10.1371/journal.pone.0294778)
Supplement: Attachment — Submitted filename: ReviewMotorchair.docx [file pone.0294778.s001.docx]

**Review “A novel motorized office chair causes low-amplitude spinal movements and activates trunk muscles: a cross-over trial”**

This paper describes a single session, repeated measures, crossover study that analyzes the effects of a novel motorized office char on muscle activity as well as spinal posture and oxygen intake. In brief, the authors noted low changes in spinal posture and some muscle activation.

It is a well designed study, yet there some methodical flaws, that make a consideration for PLOS One questionable for this version.

First, the authors do not give detailed information about the participant’s demographics. This limits the quality of the study as it has been proven that the spinal column and especially lumbar lordosis are significantly effect by age and gender. Furthermore, by not further defining the study group it is questionable in how far the results are valid for everybody.

Second, the effect on muscle activation on the stabilization of spinal function is only assessed superficially. As the MVIC in both groups were 3%, the advantage of a more technologiccaly diversed product for everyday office life is questionable.

I recommend, in order to consider a publishing in PLOS One, that the author’s thoroughly revise their paper regarding the mentioned topics before re-submission.
