# Supplementary material for: A novel motorized office chair causes low-amplitude spinal movements and activates trunk muscles: A cross-over trial
Source: PLoS One. 2023 Dec 22;18(12):e0294778. doi: 10.1371/journal.pone.0294778 (PMC10745177; doi:10.1371/journal.pone.0294778)
Supplement: Attachment — Submitted filename: Response to Reviewers.docx [file pone.0294778.s002.docx]

***Dear Dr. Schömig,***

***We would like to thank you for the feedback on our manuscript titled “A novel motorized office chair causes low-amplitude spinal movements and activates trunk muscles: a cross-over trial” and the opportunity to re-submit and address the comments made by the two expert reviewers. Also, we would like to thank the expert reviewers for taking their time to read and comment on our manuscript; the comments were clearly the result of a thorough and very insightful review. We feel that the revisions have added to the strength of the manuscript.***

***The specific reviewer comments have been addressed below. An amended version of the manuscript has been re-submitted, where changes have been highlighted in yellow.***

***Kind Regards***

***Hendrik Schäfer in behalf of the authors***

Journal Requirements:

When submitting your revision, we need you to address these additional requirements.

1. Please ensure that your manuscript meets PLOS ONE's style requirements, including those for file naming. The PLOS ONE style templates can be found at https://journals.plos.org/plosone/s/file?id=wjVg/PLOSOne_formatting_sample_main_body.pdf and https://journals.plos.org/plosone/s/file?id=ba62/PLOSOne_formatting_sample_title_authors_affiliations.pdf

**Answer:**

*Thank you for the advice! We have carefully checked the manuscript and adapted it to the style requirements.*

2. Thank you for stating the following in the Competing Interests section:

"The chair in this study was a prototype developed and provided by the start-up company Vintus. The authors did not receive any funding to conduct the study. All authors declare, that the study is rigorously performed and transparently reported."

We note that you received funding from a commercial source: Vintus

Please provide an amended Competing Interests Statement that explicitly states this commercial funder, along with any other relevant declarations relating to employment, consultancy, patents, products in development, marketed products, etc.

Within this Competing Interests Statement, please confirm that this does not alter your adherence to all PLOS ONE policies on sharing data and materials by including the following statement: "This does not alter our adherence to PLOS ONE policies on sharing data and materials.” (as detailed online in our guide for authors http://journals.plos.org/plosone/s/competing-interests). If there are restrictions on sharing of data and/or materials, please state these. Please note that we cannot proceed with consideration of your article until this information has been declared.

Please include your amended Competing Interests Statement within your cover letter. We will change the online submission form on your behalf.

**Answer:**

*We apologize for the inaccurate wording. We have not received funding from any commercial entity. Petra Platen has received a grant from the EU within the ERDF program. Two engineers have been employed to develop the prototype. We have elaborated on this circumstance extensively and included the desired statement. The amended Competing Interests Statement was added to the cover letter.*

Review Comments to the Author:

Reviewer #1:

This paper describes a single session, repeated measures, crossover study that analyzes the effects of a novel motorized office char on muscle activity as well as spinal posture and oxygen intake. In brief, the authors noted low changes in spinal posture and some muscle activation. It is a well designed study, yet there some methodical flaws, that make a consideration for PLOS One questionable for this version.

First, the authors do not give detailed information about the participant’s demographics. This limits the quality of the study as it has been proven that the spinal column and especially lumbar lordosis are significantly effect by age and gender. Furthermore, by not further defining the study group it is questionable in how far the results are valid for everybody.

**Answer:**

*Thank you for pointing out that the participant’s characteristics did not become clear for the reader. We agree that these details are highly important for interpreting to whom the study results might be representative for. Therefore, we included a participants characteristics table. A more detailed impression of the individual participants can be found in the comprehensive table in our repository:* *https://osf.io/7szrq.
In addition, we addressed the type of study population in the limitations*:
”Participants were young, healthy, and physically active. The results found are therefore only representative of the group described”

Second, the effect on muscle activation on the stabilization of spinal function is only assessed superficially. As the MVIC in both groups were 3%, the advantage of a more technologiccaly diversed product for everyday office life is questionable. I recommend, in order to consider a publishing in PLOS One, that the author’s thoroughly revise their paper regarding the mentioned topics before re-submission.

**Answer:**

*It is important to point out the limitations of superficial assessment of muscle activation.* *Thank you for raising this point! We have taken up this limitation and pointed out the problem of muscles with complex architecture such as the lumbar erector spinae:*
Farina et al. [85] postulated some issues with sEMG measurement of the trunk muscles. They reported poor signal quality and less sensitivity of surface EMG on trunk muscles with complex architecture (e.g. LES). The main limitation of the sEMG measurement in our study was the inconsistent data for left and right LES.

*It is true that both groups had similar mean activation of the lumbar muscles. However, the number of activations is higher for the moved condition. We address these different findings in detail in the discussion.* *We appreciate your concerns about the impact on the everyday office life. Therefore, we have extended the limitations:*
Even though the number of activations for CPM was higher, the mean activation remained comparable.

Reviewer #2:

The presented manuscript describes a randomized crossover study over the timeframe of 1h on a new kind of motorized office chair and its effects on sagittal lumbar movement and posture, paraspinal muscle activity, attentional control and oxygen uptake. The manuscript is mostly well written and the topic of preventive interventions in long term-sitting is highly relevant on a societal level. The multi-parameter analysis including attentional control, oxygen uptake and trunk muscle activity and spinal posture is very interesting.

Nevertheless, there are major concerns regarding the following aspects:

1. The biomechanical reasoning for the described CPM-pattern of a lying eigth is not sufficiently described. Based on the understanding of the author of the described CPM pattern, it could potentially lead to negative effects on spinal degeneration (1-4) but a sufficient analysis is lacking:

a. A biomechanical analysis of assumed effects of this pattern on spinal movement – also in comparisation and loads e.g. through in sililco simulation is lacking
b. The lying eight most likely will induce shear stress, lateral bending and eventually axional rotation, a complex movement potentially associated with induction of enhanced degeneration
c. For induction of changes in lumbar flexion/extension without shear stress, a simple sagittal change of the angulation of the sitting surface would potentially be more efficient with fewer potentially negative effects

**Answer:**

*Thank you for your kind words and your interest in our research. Your comments are provided on a well-developed understanding of biomechanics and (patho-)physiology of the spine and add important aspects to consider in this manuscript and in subsequent studies.
Our response to comments a)-c) as follows:*

a) *We agree that a computer simulation would be helpful to determine potential loads created by different movement patterns. This study could have been done before our study. However, we consider our study as in important pilot study which informed our now ongoing research. We take your advice into account and are looking forward to conducting an in silico study to inform our research. However, we feel that this would be a separate study to conduct. Our current manuscript is rather long due to the many parameters investigated, so we would refrain from including this study in the current manuscript.*
b) *Like mentioned a lot along your review, we lack a thorough investigation of possible detrimental effects.* *Shear stress and other kinematic parameters could be assessed more thoroughly by a computer simulation (see a)) or mathematical modelling with experimental data. However, shear stress, lateral bending and axial rotation do not necessarily and directly lead to detrimental effects because they occur also in everyday life activity* *(Kripa & Kaur, 2021; Swain et al., 2020).* *Overuse of any such movement – even of low intensity –might trigger pain mechanisms such as peripheral sensitization leading to tissue overloading, e.g. sustained contractions above 5% MVC can lead to muscle fatigue (van Dieën et al., 2009) and pain (Björkstén & Jonsson, 1977) as stated by (Reeves et al., 2019). To address your concerns, we added these aspects to the “Potentially adverse effects” section. However, we agree that there is a need to quantify the relationship of spinal loads with data, which we aim to do in subsequent studies.*
c) *Thank you for sharing your idea! This protocol might be a suitable alternative when individuals with low back pain and/or when they experience problems with other protocols like the “8” we investigated in this study. We take your suggestion into account in our subsequent research!*

2. the scientific profoundness of the experimental set-up as it is lacking a relevant negative and positive control based on the 1h cross-over design. As the 1h design can be argued for the complex analysis for attentional control, oxygen uptake and EMG measurements, the measurements for lumbar posture are easily applicaple to a longer time frame and the profoundness of this data would critically improve with real-life negative control (e.g. e.g. standard office-chair with movement options) and real-life positive controls (e.g. working on a standing desk) which are already implemented in the prevetion of low back pain.

**Answer:**

*Thank you again for providing thoughts on possible research designs. Again, we value your ideas that we aim to involve in our ongoing, subsequent research. However, we hope that the reviewers end the editor agree with us, that these are subjects of new studies to conduct. The data presented in this manuscript provided some of these implications, e.g. it might be beneficial to investigate the biomechanical effects with a longer duration and add adequate controls. Thus, this study adds value to the scientific, accumulate knowledge and informs future designs.*

Minor concerns:

• The introduction would benefit from a brief description of behavioral aspects of sitting and the (not yet sufficient) understanding of different lumbar load and movement patterns and lumbar degeneration.

**Answer:**

*Thank for you suggestions to improve the introduction! We included brief introducing words at the beginning on sedentary behavior and the influence on health and wellbeing. Beside a short description of static and dynamic sitting behavior in the introduction, individual sitting behavior is discussed under the section “Physiological Benefits of CPM”.*

*We are convinced that the biomechanical aspects of sitting are important for understanding the effects of CPM. Therefore, we have included a section on the biomechanical effects of dynamic sitting in the introduction.*

• The statistical analysis with simple t-test for longitudinal recorded data in multiple subjects in a crossover-design seems a little bit simplified, biostatistical counceling is recommended.

**Answer:**

*Thank you for this advice. A colleague with background in mathematics (though, not a biostatistician) gave his feedback and approval. In our analyses, we followed the approach outlined by (Wellek & Blettner, 2012), which incorporates testing procedures for treatment and period (carry-over) effects. Since our study design is as simple as outlined in the study, we deem these tests as appropriate. Since we extracted features from the time-series data and time is treated as a discrete variable in the t-tests as condition (which is a common procedure), we also present the time-series data descriptively in figures 4 and 5 to convey important information of the qualitative impact of the moving chair.*

• Additional real-life data on CPM-Chair vs a real negative control and a positive control over an 8h workday with the epionic spine system and an actimeter would add additional value regarding the translationability of the observed effects.

**Answer:**

*Thank you for pointing out this research design as well. We agree that this study design is important and are looking forward to report in this issue in our subsequent study reports.*

• A critical discussion of potentially behavioral effects such as the “rebound effect” of a CPM chair on the working-day e.g. leading potentially to longer sitting phases etc is lacking .

**Answer:**

*We are pleased about raising the point that the behavior could be influenced by the CPM or the chair itself. We take a critical stance on this in the “Potentially Adverse Effects” section*

• A critical discussion of the potential biomechanical effects of the CPM pattern on the lumbar spine is completely lacing.

**Answer:**

*We are thankful for your advice to improve the discussion. We have added a biomechanical	explanation for the effects of the CPM. Potential adverse biomechanical effects are covered by	the corresponding section.*

• A critical discussion of potential different effects of different CPM patterns is lacking.

**Answer:**

*Thank you for pointing out the importance of an investigation and discussion of different CPM patterns. We refer to the individualization of the CPM in the discussion and focusing on one CPM pattern is mentioned as a limitation. As already mentioned, we gratefully accept your suggestion and will take it into account in future studies.*

• The following sentence in the discussion does not work: “A priori power analysis based on small pilot studies”

**Answer:**

*T**he sentence is excluded, and the information is added to the following sentence, thank you!*

• In the conclusion it should be also stated, that potential negative health effects were not evaluated.

**Answer:**

*We added your suggestion, thanks!*

Therefore I recommend major revisions with at least additional data and analysis on the potential effects of the CPM-Pattern on spinal biomechanics and loading, a critical reflection of these aspects and the above mentioned commentaries in the discussion and biostatistical counseling. Furthermore additional data on spinal posture, breaks in sitting and movement (eg measured by actometers) over a working day with real life negative and positive controls and patient reported outcome measurements regarding low back pain, well-being at the workplace, subjective fatiguabilit would ad to the scientificall profoundness of the study.

**Answer:**

*Thank you once more for your intellectual contribution! Indeed, we now receive funding for three additional years to investigate long-term effects of various CPM and also stochastic movement patterns where we plan to do field studies, investigate LBP populations and relationships with additional factors such as stress. Your comments are very helpful and partly align with our plans! Again, we hope to convince you that this study provides preliminary data which is important to inform future research and, thus, adds to the knowledge of the scientific community.*
