# Supplementary material for: A novel motorized office chair causes low-amplitude spinal movements and activates trunk muscles: A cross-over trial
Source: PLoS One. 2023 Dec 22;18(12):e0294778. doi: 10.1371/journal.pone.0294778 (PMC10745177; doi:10.1371/journal.pone.0294778)
Supplement: Attachment — Submitted filename: PONE-D-22-27895R1 - Review comments.docx [file pone.0294778.s003.docx]

Dear Editor, PlusONE

Greetings.

Thank you for the opportunity to review the paper titled “A novel motorized office chair causes low-amplitude spinal movements and activates trunk muscles: a cross-over trial”.

Article ID. PONE D- 22- 27895-R1”

I appreciate the authors’ efforts in conducting this work on Twenty office workers.

The authors studied a single session (30 min) of continuous passive motion (CPM) by a novel motorized office chair on multiple variables and found slight changes in lumbar lordosis angle.

Overall, the paper is well written, but I have a few concerns about the design, sample selection and size and methodology.

Please find my review comments below.

In introduction,

In the initial paragraphs, the authors introduced sedentary sitting behaviour and physical inactivity and its impact on health-related issues such as low back pain (LBP), diabetes and cardiovascular risks. In the round-up, they stressed the importance of dynamic sitting and loading conditions of the vertebral column. Later, they introduce the advent of motorized chairs with continuous passive motion (CPM) and its effect on LBP. The authors rationalize that less passive movement (axial rotation of 1.6° from Lengsfeld's study) is insufficient to produce a positive effect, warranting a motorized chair with a greater deflection of translatory movement. Therefore, to investigate the benefits of CPM in sitting on lumbar lordosis and trunk muscle activation (rectus abdominus and erector spinae), as well as oxygen uptake in healthy adults.

The concept of dynamic sitting and its spinal loading has already been documented elsewhere, as well as strategies to prevent and manage LBP. Please motivate me why/how a CPM-motorized chair could be another potential option for trunk muscle activity (reactive) compared to person-preferred dynamic sitting (anticipatory).

If the authors address the need to conduct this study citing the limited axial rotation of 1.6° (Lengsfeld), I believe this novel motorized chair should preferably be tested in subjects with LBP. I’m just wondering if the participants are young adults (mean age of 24.5 ± 3.6 28.6 ± 3.3 years) with moderate physical activity (IPAQ moderate 1320 ± 1478; 975 ± 749), the subject selection in this study is not well aligned with the need of the study.

Methods,

A crossover design is usually chosen when the required sample size may not be met in clinical studies. Otherwise, I’m fine with the current study design. Comparing two healthy groups (one sitting on a motor chair, another group on a stable chair) may be an option!!

Also, it’s quite difficult to interpret the findings just with one session. It’s known that immediate muscle response and muscle activity do happen when someone is sitting on a moving platform. What is more interesting is how muscle activity is when someone is seated on MC for a long time. This may have a clinical implication, particularly for preventing musculoskeletal dysfunction perspective (LBP, posture etc.).

Measuring the attention control is more like a feasibility aspect of a MC whether it disturbs the attentional ability of participants. Would it be ideal to see through long working hours!! Individuals tend to adapt to postural and gaze stability when seated/standing on a low-amplitude moving platform!! Please motivate why was oxygen uptake a measure!! Would an hour sitting on a chair demand more MET!!

The authors studied only two trunk muscle activity profile (lumbar erector spinae and m. rectus abdominus). They are the phasic muscles and postural muscles (multifidus, oblique) are the first ones to contract when there is a postural perturbation!! Therefore, it's difficult to generalize the findings!! I understand that the EMG unit should be more sensitive to pick up these myosignals. I appreciate the authors' efforts to study the trunk muscles' EMG profile.

The results, findings and discussion are well-written.

Often study weaknesses are missed opportunities, but not the limitations!!

Tables and figures are useful.

There are too many citations and reference papers can be cut short.

Overall, I liked the write-up and I appreciate authors’ efforts in conducting this work.

Best wishes.

Kind regards,

The reviewer.
