# Supplementary material for: A novel motorized office chair causes low-amplitude spinal movements and activates trunk muscles: A cross-over trial
Source: PLoS One. 2023 Dec 22;18(12):e0294778. doi: 10.1371/journal.pone.0294778 (PMC10745177; doi:10.1371/journal.pone.0294778)
Supplement: Attachment — Submitted filename: Response to Reviewers.docx [file pone.0294778.s004.docx]

***Dear Dr. Monika Błaszczyszyn,***

***We would like to thank you for the feedback on our manuscript titled “A novel motorized office chair causes low-amplitude spinal movements and activates trunk muscles: a cross-over trial” and the opportunity to re-submit and address the comments made by the two expert reviewers. Also, we would like to thank the expert reviewers for taking their time to read and comment on our manuscript; the comments were clearly the result of a thorough and very insightful review. We feel that the revisions have added to the strength of the manuscript.***

***The specific reviewer comments have been addressed below. An amended version of the manuscript has been re-submitted, where changes have been highlighted in yellow.***

***Kind Regards***

***Hendrik Schäfer in behalf of the authors***

Review Comments to the Author:

Reviewer #3: Thank you for giving me the opportunity to review this interesting paper on a novel motorized chair that gave an interesting insight into movement, energy expenditure and attention during static and dynamic sitting. While the paper makes for interesting reading, I have some concerns, mostly related to the methodology, which need to be addressed before the manuscript could be considered for publication.

**Answer:**

*Dear Reviewer #3, thank you for reviewing our manuscript and for providing valuble feedback!*

Introduction:
In the third paragraph, the authors correctly point out that there is no direct link between back pain and sitting time, possibly due to the multifaceted nature of backpain. That being said, it comes a bit as a surprise that, in the following sections, the authors seem to link most of their reasoning for the conduction of this study to back pain. Please consider rewriting as evidence suggests that there are other fields of health promotion, such as sedentary behavior, that could serve as a stronger base for the justification of this study.

**Answer:**

*Thank you for raising this point. We tried to present the current status of back pain and sitting in a neutral and objective way. This should be understood as contextualization and not as a justification for conducting the study.The reason for investigating CPM lies rather in the recommendation of variable sitting.*

In the second to last paragraph, the authors use the phrase “positive effects of a passive moved sitting”. Please spell these out and relate them to your outcome measures as it makes for somewhat cumbersome reading if these positive effects have to searched for in different parts of the manuscript.

**Answer:**

*Adding this information at the section in the introduction improves the readabilty of the manuscript.*

The outcome attention is not introduced in the introduction, therefore a justification of investigating this outcome is missing, weakening the rationale of the study.

**Answer:**

*We used the AX-CPT test for two reasons: First of all the test guarantees a highly standardized measurement situation. Second, in addition it gives us informations on the attentional control. We added the outcome to the introduction.*

Methods:

Study design:

First paragraph and in general: The outcomes should always be listed in the same order throughout the manuscript as this improves readability.

**Answer:**

*Thank you for the advise. We change the section accordingly.*

The inclusion and exclusion criteria seem very brought. For example: what does any health restriction precisely refer to? For example, if a participant had a cough, was the participant excluded? Please list them precisely to make your study more reproducible. Furthermore, some outcome measures are sensitive to anthropometrics: For example sEMG and measurements of spinal kinematics are influenced by the BMI. Was there an upper and lower limit for the BMI?

**Answer:**

*Particpants who were able and allowed to work were included. This means that a patient with a cough which would not allow them to work would have been excluded.
We added this information to the manuscript.
There was no upper or lower limit for the BMI.*

The outcome attentional control comes a bit out of the blue, kindly introduce and justify it in the introduction.

**Answer:**

*Thank you for the advise. We added the informations to the introduction.*

“The hip and knee angles were set to 90°.” How was this operationalized?

**Answer:**

*We measured the angle with a protector. The height of the chair was adjusted accordingly and smaller subjects used a footrest.*

Participants:
To which guidelines are you referring to with reference 46? This sentence is not self-explanatory.

**Answer:**

*Thank your for this advise! We added the information that we refer to the IPAQ guidelines.*

*Reference 46 is: IPAQ Research Committee. Guidelines for data processing and analysis of the International Physical Activity Questionnaire (IPAQ)-short and long forms. 2005 [cited 7 Sep 2020]. Available from: www.ipaq.ki.se.*

Motorized chair:
What was the rationale to set the movement speed to 56 mm/s.?

**Answer:**

*We choosed the maximal possible velocity of the protoype in order to produce as much as possible movements for the experiment. We added this information.*

Oxygen uptake:
were different sizes of oxygen masks available?

**Answer:**

*Yes it was. We used* *Vyaire Vyntus CPX mask in size S and M to guarantee that the “the face mask with a filter was attached airtight”.*

Trunk muscle activity:
The authors state the Myon system is reliable. to what type of reliability does this refer? Inter-rater (placement of electrodes) intrarater? this is not self-explanatory.

**Answer:**

*It refers to intra-session and inter-day testing. We added the information. The information comes from the work of Sorbie et al.:*

*Sorbie GG, Williams MJ, Boyle DW, Gray A, Brouner J, Gibson N, et al. Intra-session and Inter-day Reliability of the Myon 320 Electromyography System During Sub-maximal Contractions. Front Physiol. 2018; 9:309. doi: 10.3389/fphys.2018.00309 PMID: 29651252.*

Data processing:
“The data were smoothed with a slightly moving average” what does slightly refer to, does this refer to the sEMG moving average, the epionics or something else? If the epionics and sEMG were put in relation to each other than how were they synchronized timewise, regarding aspects such as the different measurement frequencies, possible delays or inconsistent time stamps?

**Answer:**

*The moving averages were used for both the sEMG and the Epionics dataset.
We generated timestamps by tapping a the sensors twice:*

*Therefore, one sEMG sensor was attached to one Epionics accelerometer. By tapping the sensors twice, peaks are generated that allow data synchronization.*

*Then we used the table2timetable, synchronize and retime function in Matlab to align the data.*

*All our codes can be found in our repository (*[*https://osf.io/7szrq/*](https://osf.io/7szrq/)*), which offers maximal transperency.*

“Amplitudes greater than 1° were considered for further analysis”. is 1° greater than the measurement error of the device?

**Answer:**

*In the work of Consmüller et al. is shown that the difference for the flexion between Epionics and radiography is 1°.*

“repetitive movement of the chair leads to similar positive and negative angle changes” Does this imply that they are always similar in magnitude?

**Answer:**

*One movement was defined with this approach, therefore the destribution of the positive and negative magnitude is not that relevant. In addtion, we adapted the approaches of Zemp et al. and Bontrup et al.*

“To estimate the individual total movement in each condition, the number of movements
was multiplied by the average movement amplitude“ why was it multiplied by the average, why not make a sum of all peak to peak amplitudes? kindly explain

**Answer:**

*Thank you for this idea. This would be also a good and probaply a more precise way to report the total movement. However, our intention was to combine the given information, which are in the variables “number of movements” and “mean amplitude [°]”, to make the difference in the conditions more descriptive. We believe that outcome would not be influenced by the two calculation methods.*

“The sEMG and Epionics data were synchronized to assess the spinal movements and their corresponding muscle activity.” how often were they synchronized? How many recordings were done per participant and how did you handle the manual synchronization given the two different recording frequencies? did you upsample or downsample one of the time series?
“By tapping the sensors twice, peaks are generated that allow data synchronization.” First, I would describe this in the beginning of this section as it is important to understand how you performed your measurements. second please explain why this method is valid considering that you have different recording frequencies, also in light of other potential sources of bias such as delays in recordings.

**Answer:**

*The measurements were synchronized once in the beginning.
There was only one recording for each participant.
We used the table2timetable, synchronize and retime functions in Matlab. The scripts can be found in our repository.
We believe that is essential to describe the processing of the data before the synchronization is mentioned.
We checked if the method is valid by evaluating the timepoints of the on- and offset of the conditions, in order to account for delays in recordings.*

*Of note, the analysis of our data and the causal connection confirms that this method is valid (Figure 4).*

Statistical analysis:

you did test for the normality of the data distribution so what was your rationale for the t-tests? also explain your reasoning for using an unpaired t-test in light of the argument that Paired t-test is used to compare the means of two samples when each individual in one sample also appears in the other sample.

“Non-normally distributed data were analyzed unchanged.” please explain why you checked the distribution if it had no consequence? what is your rationale for not switching to non-parametric tests?

**Answer:**

*We strictly followed the instructions of Wellek and Blettner (2012):*

Wellek S, Blettner M. On the proper use of the crossover design in clinical trials: part 18 of a series on evaluation of scientific publications. Deutsches Ärzteblatt International. 2012; 109:276–81. doi: 10.3238/arztebl.2012.0276 PMID: 22567063.

*The explanation for using unpaired t tests can be found in the work of Wellek and Blattner:*

“The differences between treatment effects can be assessed by means of a standard t-test for independent samples using the intra-individual differences between the outcomes in both periods as the raw data.”

*We calculated and reported the distribution in order to give this information to reader.
We did not switched to nonparametric tests, to stay inline with the recommendations of Wellek and Blattner.
The data and the calculations can be found in our repository.*

Results:
consider using the same order of outcomes in each section, as this greatly helps the reader.

**Answer:**

*The order is:*

- *Methods
  Attentional control, Oxygen uptake, Trunk muscle activity, Spinal posture*
- *Results
  Attentional control, Oxygen uptake, Trunk muscle activity, Spinal posture,*
- *Discussion
  Attentional control, Energy expenditure, Muscle activity, Posture*

Trunk muscle activity
“Data inconsistencies occurred quite regularly (e.g., large differences between left and right side, low signal), but we did not exclude these data.” why not, what was your rationale for not considering these as measurement errors or "bad data"? how do you justify this given the assumption that these might influence your results and perhaps lead you to wrong conclusions?

**Answer:**

*We discussed this with our research team and concluded that we cannot assess whether this is physiological or due to measurement error. Therefore, we decided not to exclude the data. We are confident that this does not affect our conclusions as the discrepancies existed in both conditions.*

Spinal posture:
Figure 5b shows that the static movement is rather random, compared to the cyclic movement in 5a. can you justify that the methods used to derive peak to peak amplitude in a cyclic movement are also valid for the more random movement observed in 5b? How do you explain that the three subjects in figure 5 show a very different basic lordosis angle, can this be due to placement or measurement error?
how does the small range of motion compare to the measurement error or placement error?

**Answer:**

*It is justified by the way of processing the data. When the difference between two amplitudes was greater than 1° it was count as one movement. In addition, we conducted visual checks if all movements were detected. The codes for the figures can also be found in our scripts.*

*“The mean lumbar lordosis angle was highly variable for MC 0.3° ± 14.9° and for SC 2.8° ± 15.4° (p = .058; d = .165).”
We argue:*
*“Our findings show large deviations of the mean lumbar lordosis angle, which could be attributed to the different anatomically shaped lordosis (Fig. 5).”
Therefore, we do not consider placement or measurement errors as reasons for this difference.*

Discussion
Attentional control:
“After the removal of an outlier, the average performance for MC was 1 % higher(p = .059).» is this a relevant difference, also regarding intra-subject variance and all other factors that can influence attentional control that you might not have been able to control?

**Answer:**

*No, however, we argue that the hypothesis from Lengsfeld et al. can not be confirmed with our observered results. Therefore we just state that there is no difference between the two conditions and hence no disadvantage of the moved conditions.*

Muscle activity
“Therefore, movements in dynamic sitting are caused by muscle activation.” sentence seems misleading, if meaning is that only motorized seats can cause additional muscle activation, kindly refer to https://pubmed.ncbi.nlm.nih.gov/35905083/ (pelvis initiated exercises)
Otherwise kindly explain if you imply that the muscular activity could not also be reactive to the moving seat.

**Answer:**

*We just wanted to state that movements in dynamic sitting are caused by the use of muscle, whereas the CPM of the motorized chair is responsible for the movement. Since the CPM causes the movement the muscle activity is likely reactive to the moving seat.*

¨The m. rectus abdominus (REC) was almost inactive during both conditions.» was it inactive or was the activity not detectable?
“Segmental stability is considered an important factor in back pain prevention.” given the heterogeneity of LBP, is this statement a bit too brave? can you elaborate on which type of LBP sufferers might benefit from active sitting?

**Answer:**

*We conclude that the muscle was almost inactive because: “*Less activity of the abdominal muscles is assumed when sitting. (O'Sullivan et al. 2006)”.
*We deleted the “important” in order to account for the heterogeneity of LBP.
We discuss this in the benefit section:*
“(…)This behavior is typical for people with LBP. They show more frequent static, end-range postures with less small and more large infrequent movements [83,84]. The chair could be useful for people with static sitting behavior and/or LBP.”

Posture
“The CPM did not affect the spinal posture regarding the lumbar lordosis angle, which did not differ for the two conditions.” With the information provided in Figure 5: the mean angle did not differ much, but the angular displacement is very different (cyclic vs random movement).
“Our findings show large deviations of the mean lumbar lordosis angle, which could be attributed to the different anatomically shaped lordosis (Fig. 5).” this could be verified by investigating neutral sitting or standing, are there data on neutral sitting perhaps from epionics or from video? if yes, kindly verify this assumption as it would also help to ensure that this not due to a measurement or placement error.

**Answer:**

*We did not assessed this. However we cite 5 articles from the research groups of Pries and Taylor were this data are available. For example the work of Consmüller (*doi: 10.1007/s00586-012-2312-1) *shows also different anatomically shaped lordosis and confirms the observation with other measurements (radiography).*

Physiological benefits of CPM
“They show more frequent static, end-range postures with less small and more large
infrequent movements « Figure 5: ID04 does not look like an end of range posture, how many participants did assume an end range posture?

**Answer:**

*It is right that participants with ID4 does not show an end range posture. We were rather referring to more frequent static, less small and more large infrequent movements.
We can not give any informations on end range posture since this was not assessed.*

“A screening of the user would enable individualized CPM.” while this is an interesting thought, this is nevertheless a frequent and often unproven claim made for any novel health technology. how should an individualized CPM be derived from screening of which parameters?

**Answer:**

*These are interesting questions that could be investigated in further studies. Our work provides a starting point for these considerations. In particular, the various patterns in Figure 5 are helpful for examining this.*

Movement counts:
“In addition, the interaction between muscles and movements can be assessed. The highest muscle activity is observed when LES is in the most approximated position. The difference between the number of movements (498) and activations (~203) could be explained by only one peak activation during a cycle.” A graph illustrating this would be very interesting, however given the methodological reservation expressed earlier it first needs to be explained by the authors how they synchronized the time series in more detail.

**Answer:**

*The information of the difference between the number of movements and activations is in the figure 4. In 4.a could only one clear peak be detected whereas in 4.b two peaks are shown.
The synchronization was done with the matlab table2timetable, synchronize and retime function with an linear interpolation of the epionics data, due to the lower sampling rate.*

Reviewer #4: Dear Authors,

Greetings.
Thank you for the opportunity to review the paper titled “A novel motorized office chair causes low-amplitude spinal movements and activates trunk muscles: a cross-over trial”.

Article ID. PONE D- 22- 27895-R1”

I appreciate the authors’ efforts in conducting this work on Twenty office workers.
The authors studied a single session (30 min) of continuous passive motion (CPM) by a novel motorized office chair on multiple variables and found slight changes in lumbar lordosis angle.

**Answer:**

*Dear Prof. Dr. Karthikbabu, thank you for your kind words and your interest in our research. We appreciate your valuable feedback and we try to answer all of your questions.*

Overall, the paper is well written, but I have a few concerns about the design, sample selection and size and methodology.

Please find my review comments below.
In introduction,
In the initial paragraphs, the authors introduced sedentary sitting behaviour and physical inactivity and its impact on health-related issues such as low back pain (LBP), diabetes and cardiovascular risks. In the round-up, they stressed the importance of dynamic sitting and loading conditions of the vertebral column. Later, they introduce the advent of motorized chairs with continuous passive motion (CPM) and its effect on LBP. The authors rationalize that less passive movement (axial rotation of 1.6° from Lengsfeld's study) is insufficient to produce a positive effect, warranting a motorized chair with a greater deflection of translatory movement. Therefore, to investigate the benefits of CPM in sitting on lumbar lordosis and trunk muscle activation (rectus abdominus and erector spinae), as well as oxygen uptake in healthy adults.

The concept of dynamic sitting and its spinal loading has already been documented elsewhere, as well as strategies to prevent and manage LBP. Please motivate me why/how a CPM-motorized chair could be another potential option for trunk muscle activity (reactive) compared to person-preferred dynamic sitting (anticipatory).

**Answer:**

*Thank you for raising this point! We are addressing this point in the discussion (Physiological benefits of CPM). In general, whenever there is an voluntary muscle contraction then this is superior to the reactive activation of muscle caused by the CPM. Therefore, it could be assumed that individuals with dynamic sitting behavior do not have the need for CPM then others with static sitting behavior.To conclude, CPM can be beneficial for individuals with static sitting behavior and low personal willingness to change their behavior.*

If the authors address the need to conduct this study citing the limited axial rotation of 1.6° (Lengsfeld), I believe this novel motorized chair should preferably be tested in subjects with LBP. I’m just wondering if the participants are young adults (mean age of 24.5 ± 3.6 28.6 ± 3.3 years) with moderate physical activity (IPAQ moderate 1320 ± 1478; 975 ± 749), the subject selection in this study is not well aligned with the need of the study.

**Answer:**

*We are thankful for this consideration, since we are planning to investigate this in our next studies.
However, this is the first study of this novel chair and we decided to first evaluate the effects of the CPM on healthy individuals. Another minor reason was that due to Covid-19 restrictions, only employees in our department could be tested.*

Methods,

A crossover design is usually chosen when the required sample size may not be met in clinical studies. Otherwise, I’m fine with the current study design. Comparing two healthy groups (one sitting on a motor chair, another group on a stable chair) may be an option!!

**Answer:**

*Yes, this is totally right. However, we decided to use the single session design, in order to have the less potantially influencing factors in both conditions.*

Also, it’s quite difficult to interpret the findings just with one session. It’s known that immediate muscle response and muscle activity do happen when someone is sitting on a moving platform. What is more interesting is how muscle activity is when someone is seated on MC for a long time. This may have a clinical implication, particularly for preventing musculoskeletal dysfunction perspective (LBP, posture etc.).

**Answer:**

*This is definitley an interesting aspect and in scope of our further investigations. However, we state in the section “Muscle Activity” that “(..) long-term effects have not yet been studied and are therefore uncertain.”*

Measuring the attention control is more like a feasibility aspect of a MC whether it disturbs the attentional ability of participants. Would it be ideal to see through long working hours!!

**Answer:**

*We cannot say that MC is in favor regarding the attentional control, but we were also surprised by the results. It would be definitely interesting to investigate longer duration, especially when the results of the work of Henz et al is taken into account:
“They reported an improvement in mathematical performance during dynamic sitting”
“Henz et al. [68] observed longer durations of up to 4 hours (…)”*

Individuals tend to adapt to postural and gaze stability when seated/standing on a low-amplitude moving platform!! Please motivate why was oxygen uptake a measure!! Would an hour sitting on a chair demand more MET!!

**Answer:**

*We had the question if CPM would increase the demand over the treshold (MET ≥ 1.5) for sedentary behaviour, which was not confirmed. We argue that longer duration of CPM would not increase the oxygen uptake, since we could not detect an increase with the observerd 20 minutes.*

The authors studied only two trunk muscle activity profile (lumbar erector spinae and m. rectus abdominus). They are the phasic muscles and postural muscles (multifidus, oblique) are the first ones to contract when there is a postural perturbation!! Therefore, it's difficult to generalize the findings!! I understand that the EMG unit should be more sensitive to pick up these myosignals. I appreciate the authors' efforts to study the trunk muscles' EMG profile.

**Answer:**

*It is right that it is difficult to assess the muscle activity with the sEMG. Therefore we tried to interpret the results with coution and added a critical discussion citing relevant articles such as the one of Farina et al. Thank you very much for honoring our work!*

The results, findings and discussion are well-written.

Often study weaknesses are missed opportunities, but not the limitations!!
Tables and figures are useful.

There are too many citations and reference papers can be cut short.
Overall, I liked the write-up and I appreciate authors’ efforts in conducting this work.
Best wishes.

Kind regards,
The reviewer.
